# *De novo *characterization of a whitefly transcriptome and analysis of its gene expression during development

**DOI:** 10.1186/1471-2164-11-400

**Published:** 2010-06-24

**Authors:** Xiao-Wei Wang, Jun-Bo Luan, Jun-Min Li, Yan-Yuan Bao, Chuan-Xi Zhang, Shu-Sheng Liu

**Affiliations:** 1 Ministry of Agriculture Key Laboratory of Molecular Biology of Crop Pathogens and Insects, Institute of Insect Sciences, Zhejiang University, Hangzhou 310029, China

## Abstract

**Background:**

Whitefly (*Bemisia tabaci*) causes extensive crop damage throughout the world by feeding directly on plants and by vectoring hundreds of species of begomoviruses. Yet little is understood about its genes involved in development, insecticide resistance, host range plasticity and virus transmission.

**Results:**

To facilitate research on whitefly, we present a method for *de novo *assembly of whitefly transcriptome using short read sequencing technology (Illumina). In a single run, we produced more than 43 million sequencing reads. These reads were assembled into 168,900 unique sequences (mean size = 266 bp) which represent more than 10-fold of all the whitefly sequences deposited in the GenBank (as of March 2010). Based on similarity search with known proteins, these analyses identified 27,290 sequences with a cut-off E-value above 10^-5^. Assembled sequences were annotated with gene descriptions, gene ontology and clusters of orthologous group terms. In addition, we investigated the transcriptome changes during whitefly development using a tag-based digital gene expression (DGE) system. We obtained a sequencing depth of over 2.5 million tags per sample and identified a large number of genes associated with specific developmental stages and insecticide resistance.

**Conclusion:**

Our data provides the most comprehensive sequence resource available for whitefly study and demonstrates that the Illumina sequencing allows *de novo *transcriptome assembly and gene expression analysis in a species lacking genome information. We anticipate that next generation sequencing technologies hold great potential for the study of the transcriptome in other non-model organisms.

## Background

The whitefly *Bemisia tabaci *(Gennadius) is a genetically diverse complex containing some of the most destructive invasive pests of many ornamental and glasshouse crops worldwide [1,2]. The species complex colonizes more than 600 different species of plants, transmits many plant viruses, feeds on phloem sap, and promotes the growth of damaging fungi on honeydew excretions deposited on plants [3-6]. Recent phylogenetic analysis combined with a pattern of reproductive isolation among genetic groups within *B. tabaci *indicate that the complex contains at least 24 cryptic species, some of which have been referred to as "biotypes" in the last 20 years [7,8]. As the separation at the species level within the *B. tabaci *complex is yet to be fully resolved, we have retained the commonly used term "biotype" to link this study with existing literature. The most predominant and damaging biotypes of *B. tabaci *are the B and Q biotypes [9,10]. While the former is known for its high fitness parameters, the Q biotype whitefly has a unique ability to develop and maintain high levels of resistance to major classes of insecticides owing to biological and genetic factors [11,12].

Despite its global importance, genomic sequence resources available for the whitefly are scarce, especially for the Q biotype. Currently (March 30th, 2010), there are about 9110 EST and 762 nucleotide sequences available on NCBI for the B biotype whitefly, and only 683 nucleotide sequences have been deposited for the Q biotype whitefly. The previous EST sequencing efforts for the B biotype whitefly have allowed the development of small-scale microarrays for gene expression analysis in the context of insecticide resistance and parasitoid-whitefly interactions [13-15]. While these studies have highlighted the utility of cDNA sequencing for candidate gene discovery in the absence of a genome sequence, a comprehensive description of the genes expressed in insecticide-resistant Q biotype whitefly remains unavailable.

Over the past several years, the next generation sequencing technology has emerged as a cutting edge approach for high-throughput sequence determination and this has dramatically improved the efficiency and speed of gene discovery [16,17]. For example, the Illumina sequencing technology is able to generate over one billion bases of high-quality DNA sequence per run at less than 1% of the cost of capillary-based methods [18]. Furthermore, this next generation sequencing has also significantly accelerated and improved the sensitivity of gene-expression profiling and, is expected to boost collaborative and comparative genomics studies [19,20]. Previously, Illumina sequencing of transcriptomes for organisms with completed genomes confirmed that the relatively short reads produced can be effectively assembled and used for gene discovery and comparison of gene expression profiles [21,22]. Despite its obvious potential, next generation sequencing methods have not yet been applied to whitefly research.

In this study, we generated over three billion bases of high-quality DNA sequence with Illumina technology and demonstrated the suitability of short-read sequencing for *de novo *assembly and annotation of genes expressed in a eukaryote without the prior genome information. In a single run, we identified 168,900 distinct sequences including hundreds of insecticide target and metabolism genes. Furthermore, we compared the gene expression profiles of whiteflies during different developmental stages using a digital gene expression system. The assembled, annotated transcriptome sequences and gene expression profiles provide an invaluable resource for the identification of whitefly genes involved in insecticide resistance, development and virus transmission.

## Results and discussion

### Illumina sequencing and reads assembly

To obtain an overview of the whitefly gene expression profile at different developmental stages, a cDNA sample was prepared from whitefly eggs, nymphs, pupae and adults and sequenced using the Illumina sequencing platform. After cleaning and quality checks, we obtained 43 million of 75 bp reads from one plate (8 lanes) of sequencing. To facilitate sequence assembly, these raw reads were randomly clipped into 21-mers for sequence assembly using SOAPdenovo software [23]. These short 21-mers were assembled, resulting in 4,274,766 contigs (Table 1). The mean contig size was 40 bp with lengths ranging from as small as 22 bp to as large as 2,189 bp. The size distribution of these contigs is shown in Additional file 1. Using paired-end joining and gap-filling, the contigs were further assembled into 170,115 scaffolds with a mean size of 266 bp including 4,588 scaffolds larger than 1,000 bp (Table 1). After clustering using TGICL software [24], the 170,115 scaffolds generated 168,900 distinct sequences with 1,206 clusters (mean size: 372 bp) and 167,694 singletons (mean size: 265 bp) (Table 1). In this manuscript, the singleton means a scaffold that matches no other scaffolds and the distinct sequences include all the clusters and singletons generated from scaffolds. The size distribution of these distinct sequences is shown in Additional file 1. To demonstrate the quality of sequencing data, we randomly selected 6 singletons and designed 6 pairs of primers for RT-PCR amplification. In this analysis, 5 out of 6 primer pairs resulted in a band of the expected size and the identity of all five PCR products were confirmed by Sanger sequencing (data not shown).

**Table 1:** Summary for the Q biotype whitefly transcriptome

Total number of reads	43,731,400
Total base pairs (bp)	3,279,855,000
Average read length	75 bp
Total number of contigs	4,274,766
Mean length of contigs	40 bp
Total number of scaffolds	170,115
Mean length of scaffolds	266 bp
Distinct clusters	1,206
Distinct singletons	167,694
Total distinct sequences	168,900
Sequences with E-value < 10^-5^	27,290

### Annotation of predicted proteins

**Figure 1:**
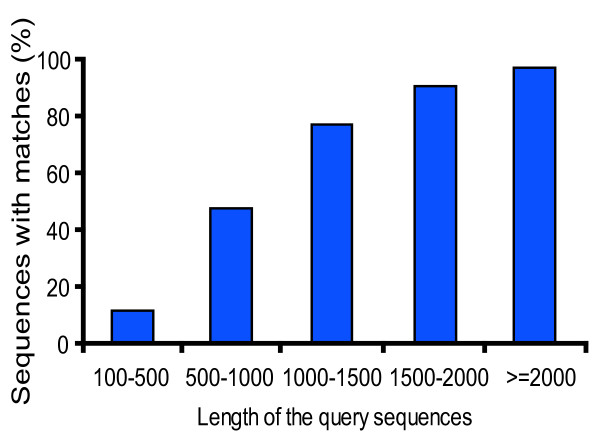
**Effect of query sequence length on the percentage of sequences for which significant matches were found**. The proportion of sequences with matches (with a cut-off E-value of 1.0E-5) in NCBI nr databases is greater among the longer assembled sequences.

**Figure 2:**
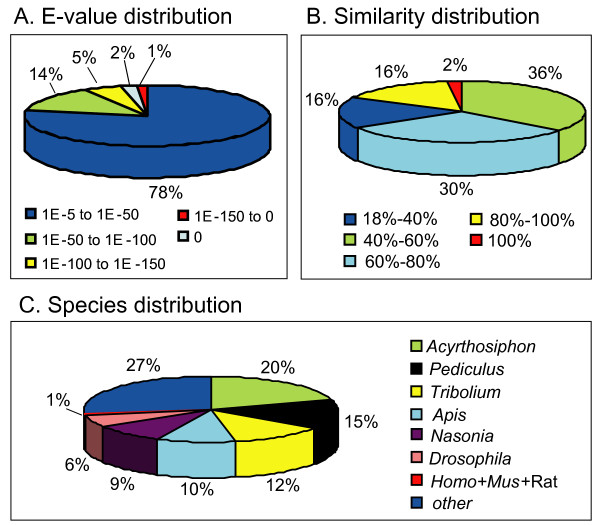
**Characteristics of homology search of Illumina sequences against the nr database**. (A) E-value distribution of BLAST hits for each unique sequence with a cut-off E-value of 1.0E-5. (B) Similarity distribution of the top BLAST hits for each sequence. (C) Species distribution is shown as a percentage of the total homologous sequences with an E-value of at least 1.0E-5. We used the first hit of each sequence for analysis. Homo: *Homo sapiens*; Mus: *Mus musculus*; Rat: *Rattus norvegicus*.

For annotation, distinct gene sequences were first searched using BLASTx against the non-redundant (nr) NCBI nucleotide database using a cut-off E-value of 10^-5^. Using this approach, 27,290 genes (16.2% of all distinct sequences) returned an above cut-off BLAST result (see Additional file 2). Because of the relatively short length of distinct gene sequences (mean size of 266 bp) and lack of genome information in whitefly, most of the 168,900 assembled sequences could not be matched to known genes (83.8%). Figure 1 indicates that the proportion of sequences with matches in nr databases is greater among the longer assembled sequences. Specifically, a 97% of match efficiency was observed for sequences longer than 2,000 bp, whereas the match efficiency decreased to about 48% for those ranging from 500 to 1,000 bp and to 11.5% for sequences between 100 to 500 bp (Figure 1). The E-value distribution of the top hits in the nr database showed that 22% of the mapped sequences have strong homology (smaller than 1.0E^-50^), whereas 78% of the homolog sequences ranged between 1.0E^-5 ^to 1.0E^-50 ^(Figure 2A). The similarity distribution has a comparable pattern with 18% of the sequences having a similarity higher than 80%, while 82% of the hits have a similarity ranging from 18% to 80% (Figure 2B). For species distribution, 20% of the distinct sequences have top matches (first hit)trained with sequences from the hemipteran species pea aphid (*Acyrthosiphon pisum*), followed by the body louse (*Pediculus humanus corporis*) (15%), red flour beetle (*Tribolium castaneum*) (12%) and honey bee (*Apis mellifera*) (10%) (Figure 2C). There were 126 distinct sequences with the highest homology to genes from *B. tabaci *and the majority of these hits were to cytochrome P450 and heat shock protein (data not shown).

### Gene ontology (GO) and clusters of orthologous groups (COG) classification

**Figure 3:**
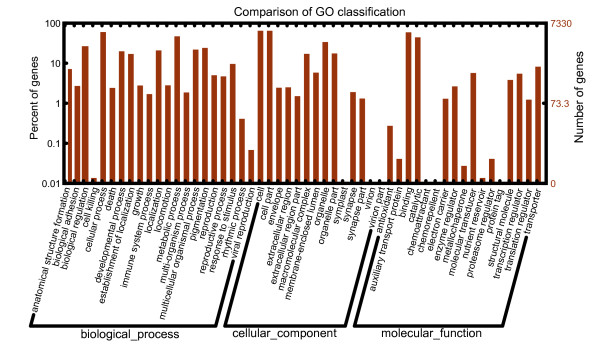
**Histogram presentation of Gene Ontology classification**. The results are summarized in three main categories: biological process, cellular component and molecular function. The right y-axis indicates the number of genes in a category. The left y-axis indicates the percentage of a specific category of genes in that main category.

**Figure 4:**
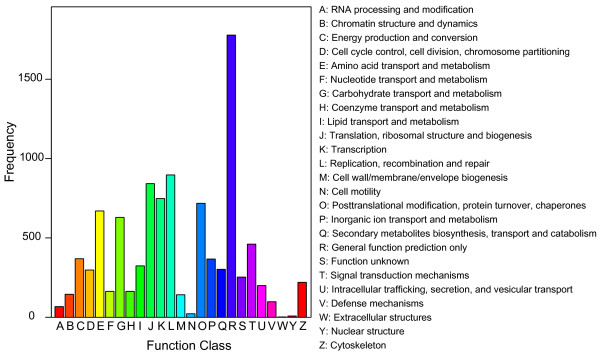
**Histogram presentation of clusters of orthologous groups (COG) classification**. Out of 27,290 nr hits, 7790 sequences have a COG classification among the 25 categories.

GO assignments were used to classify the functions of the predicted Q biotype whitefly genes. Based on sequence homology, 7,330 sequences can be categorized into 52 functional groups (Figure 3). In each of the three main categories (biological process, cellular component and molecular function) of the GO classification, 'Cellular process', "Cell part" and "Binding" terms are dominant respectively; however, we did not find any genes in the clusters of 'Symplast', 'Chemoattractant' and 'Chemorepellent'. We also noticed a high-percentage of genes from categories of 'Metabolic process', 'Catalytic' and 'Pigmentation' and only a few genes from terms of 'Cell killing' and 'Nutrient reservoir' (Figure 3). To further evaluate the completeness of our transcriptome library and the effectiveness of our annotation process, we searched the annotated sequences for the genes involved in COG classifications. In total, out of 27,290 nr hits, 7,790 sequences have a COG classification (Figure 4). Among the 25 COG categories, the cluster for 'General function prediction' represents the largest group (1778, 22.8%) followed by 'Replication, recombination and repair' (897, 11.5%) and 'Translation, ribosomal structure and biogenesis' (842, 10.8%). The following categories: extracellular structures (2, 0.025%); nuclear structure (8, 0.1%) and cell motility (22, 0.28%), represent the smallest groups (Figure 4). To identify the biological pathways that are active in the Q biotype whitefly, we mapped the 27,290 annotated sequences to the reference canonical pathways in Kyoto Encyclopedia of Genes and Genomes (KEGG) [25]. In total, we assigned 11,104 sequences to 214 KEGG pathways. The pathways with most representation by the unique sequences were starch and sucrose metabolism (553 members); purine metabolism (458 members) and galactose metabolism (183 members). These annotations provide a valuable resource for investigating specific processes, functions and pathways during whitefly research.

### Detection of sequences related to the insecticide targets and metabolism

As the Q biotype whitefly is highly resistant to insecticides, we analyzed the sequences related to insecticide targets and metabolism and compared our data with sequences from NCBI nucleotides and EST database [13]. As shown in Table 2, we identified a number of sequences which are homologous to enzymes related to insecticide metabolic resistance, such as carboxylesterase and glutathione S-transferase; and insecticide targets, such as acetylcholinesterase, GABA receptor and nicotinic acetylcholine receptor. Among them, the GABA receptor sequences were identified from the whitefly for the first time. In total, we obtained 28 nicotinic acetylcholine receptor related sequences. After removing redundant sequences, we identified 9 different nicotinic acetylcholine receptor subunits (Table 3). As an insect genome generally has 10 to 11 nicotinic acetylcholine receptor subunits [26], our database represents a nearly complete collection of such genes in whitefly. This finding further demonstrates the quality of our sequencing data and will certainly facilitate Q biotype whitefly insecticide resistance research. It was previously observed that the resistance to organophosphates in the B biotype *B. tabaci *was associated with a point mutation (Phe392Trp) in *ace1*-type acetylcholinesterase [27]. Therefore, we compared the protein sequence of the Q biotype whitefly *ace1*-type acetylcholinesterase with that of the B biotype. Sequence alignment shows that the Q biotype whitefly acetylcholinesterase also contains the Phe392Trp mutation (data not shown). Furthermore, three additional mutations were observed in the N-terminal region of the Q biotype whitefly *ace1*-type acetylcholinesterase (Additional file 3) which might confer further organophosphate resistance. However, the relevance of these additional mutations requires further investigation.

**Table 2:** Genes related to the insecticide targets and metabolism

Gene name	Number of sequences had a hit with nr database^1^	Number of known sequences from NCBI nucleotide database^2^	**Number of known sequences from B biotype whitefly EST project **[13]
Carboxylesterase	14	9	0
Catalase	4	0	0
Cytochrome P450	184	17	21
Glutathione S-transferase	14	0	7
NADH dehydrogenase	26	3	749
NADH oxidoreductase	7	0	112
Trypsin	13	0	5
Superoxide dismutase	6	0	10
Acetylcholinesterase	5	7	0
GABA receptor	2	0	0
Nicotinic acetylcholine receptor	28	2	4
Sodium channel	21	16	0

^1^Number of sequences obtained in this study that had a hit with the corresponding proteins in the NCBI nr database.

^2^Number of sequences from NCBI nr database that show homology with corresponding proteins (as of March 2010).

**Table 3:** Identified nicotinic acetylcholine receptor (nAChR) genes

nAChR subunits	Gene ID	Length	Subject ID	Species	E value
alpha 1 subunit	Singletons162135	464	NP_001091690.1	*Apis mellifera*	5.00E-83
alpha 2 subunit	Singletons18288	846	AAD09808.1	*Heliothis virescens*	1.00E-122
alpha 3 subunit	Singletons14592	2136	CAI54098.1	*Bemisia tabaci*	0
alpha 4 subunit	Singletons1403	1044	ABE67099.1	*Bemisia tabaci*	1.00E-176
alpha 5 subunit	Singletons163989	539	ACM09845.1	*Tribolium castaneum*	3.00E-63
Nla6 subunit	Singletons43497	103	ACL14949.1	*Nilaparvata lugens*	2.00E-09
alpha 7 subunit	Singletons117529	170	ABV72697.1	*Tribolium castaneum*	5.00E-25
alpha 10 subunit	Singletons7258	273	ACP31313.1	*Tribolium castaneum*	5.00E-12
beta 1 subunit	Singletons17694	1134	ACJ07013.1	*Nilaparvata lugens*	1.00E-146

### Digital gene expression (DGE) library sequencing

**Figure 5:**
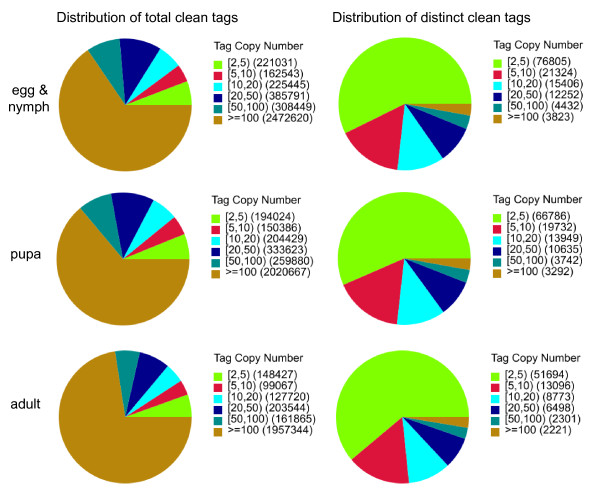
**Distribution of total tags and distinct tags over different tag abundance categories**. (A) Distribution of total tags. Numbers in the square brackets indicate the range of copy numbers for a specific category of tags. For example, [2,5] means all the tags in this category has 2 to 5 copies. Numbers in the parentheses show the total tag copy number for all the tags in that category. (B) Distribution of distinct tags. Numbers in the square brackets indicate the range of copy numbers for a specific category of tags. Numbers in the parentheses show the total types of tags in that category.

An immediate application of our transcriptome sequence data includes gene expression profiling under different physiological conditions. Using the DGE method, which generates absolute rather than relative gene expression measurements and avoids many of the inherent limitations of microarray analysis, we analyzed the gene expression variations during whitefly development. We sequenced three DGE libraries: egg & nymph, pupa and adult, and generated between 2.8 and 3.9 million raw tags for each of the three samples (Table 4). After removing the low quality reads, the total number of tags per library ranged from 2.7 to 3.8 million and the number of tag entities with unique nucleotide sequences ranged from 84,583 to 134,042 (Table 4). For mRNA expression, heterogeneity and redundancy are two significant characteristics. While the majority of mRNA is expressed at low levels, a small proportion of mRNA is highly expressed. Therefore, the distribution of tag expression was used to evaluate the normality of the DGE data. As shown in Figure 5, the distribution of total tags and distinct tags over different tag abundance categories showed similar patterns for all three DGE libraries. However, under the distribution of total tags, high-expression tags with copy numbers larger than 100 are in absolute dominance whereas low-expression tags with copy numbers smaller than 10 occupy the majority of distinct tag distributions (Figure 5). To evaluate the reproducibility of DGE library sequencing, we performed Pearson correlation analysis for pupa vs. adult and egg & nymph vs. pupa (Additional file 4). Pearson correlations for the parallel libraries were 0.96 and 0.99 respectively, indicating the reliability of our experimental results as well as operational stability.

**Table 4:** Statistics of DGE sequencing

Summary			egg & nymph	pupa	adult
Raw Tag	Total		3,936,335	3,310,027	2,812,938
Raw Tag	Distinct		276,610	251,073	183,449
Tag	Total Tag	number	3,775,879	3,163,009	2,697,967
Tag	Distinct Tag	number	134,042	118,136	84,583
Tag Mapping to Gene	Total	number	965,899	820,877	508,192
Tag Mapping to Gene	Total	% of tag	25.58%	25.95%	18.84%
Tag Mapping to Gene	Distinct Tag	number	73,555	68,714	46,049
Tag Mapping to Gene	Distinct Tag	% of tag	54.87%	58.17%	54.44%
Tag-mapped Genes		number	38,548	37,045	26,998
Tag-mapped Genes		% of ref genes	22.82%	21.93%	15.98%

### Mapping sequences to the reference transcriptome database

**Figure 6:**
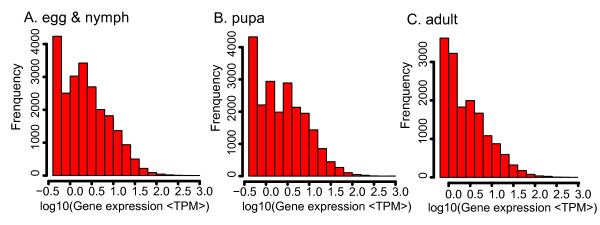
**The level of gene expression for each gene**. Gene expression level was determined by calculating the number of unambiguous tags for each gene and then normalizing to TPM (transcript copies per million tags).

To reveal the molecular events behind DGE profiles, we mapped the tag sequences of the three DGE libraries to our transcriptome reference database generated in the above-mentioned Illumina sequencing. This reference database contains 168,900 distinct sequences with 159,671 unambiguous reference tags. Among the 84,583 to 134,042 distinct tags generated from the Illumina sequencing of the three libraries, 46,049 to 73,555 distinct tags were mapped to a gene in the reference database (Table 4). Tags mapped to a unique sequence are the most critical subset of the DGE libraries as they can explicitly identify a transcript. Up to 22.82% (38,548) of the sequences in our transcriptome reference tag database could be unequivocally identified by unique tag (Table 4). To confirm whether the number of detected genes increases proportionally to sequencing amount (total tag number), a saturation analysis was performed. Additional file 5 shows a trend of saturation where the number of detected genes almost ceases to increase when the number of reads reaches 2 million. Next, the level of gene expression was determined by calculating the number of unambiguous tags for each gene and then normalizing this to the number of transcripts per million tags (TPM). As summarized in Figure 6, the results show that mRNA transcribed from the major kinds of genes is represented in fewer than ten copies and only a small proportion of genes are highly expressed. In the Additional file 6, we have listed the top 20 most abundantly expressed genes from adult whiteflies. While analyzing the five most abundantly expressed genes with annotation, we noticed the presence of glyceraldehyde-3-phosphate dehydrogenase, translation elongation factor 2, cytochrome P450 CYP6CX1v2 and tubulin alpha chain in all the three libraries.

### Distribution of DGE tags on genes

DGE is a SAGE-based transcript profiling method. In theory, all the sequenced tags in the libraries should be mapped to the last NlaIII restriction site on the sense strand at the 3'-end of the transcript. However, like the results in DGE analysis of mouse and zebrafish transcriptomes [20,22], we found that approximately 60% of the tags mapped to the classical site (data not shown). This is probably due to the incomplete NlaIII digestion during library preparation and the usage of alternative polyadenylation and/or splicing sites [28]. Detection of multiple tags with high count numbers for a predicted transcript indicates the reliability of the transcript sequence [22]. Furthermore, the information obtained from multiple tags per transcript is valuable for the verification of *ab initio *gene predictions.

### Changes in gene expression profile among the different developmental stages

**Figure 7:**
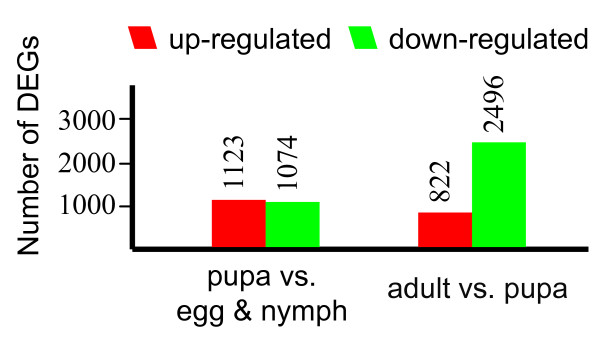
**Changes in gene expression profile among the different developmental stages**. The number of up-regulated and down-regulated genes between pupa and egg & nymph; adult and pupa are summarized.

To identify genes showing a significant change in expression during different developmental stages, the differentially expressed tags between two samples were identified by an algorithm developed by Audic *et al. *[29]. A total of 12,257 significantly changed tag entities were detected between the adult and pupa whitefly libraries. Those tags were mapped to 3,318 genes with 822 genes up-regulated and 2,496 genes down-regulated (Figure 7A & Additional file 7). Between pupa and egg & nymph libraries, a total of 2,197 differentially expressed genes were detected with roughly the same amount of up-regulated genes (1,123) and down-regulated genes (1,074) (Additional file 8). This suggests that the number of differentially expressed genes between pupa and adult are larger than that between pupa and egg & nymph. Next we analyzed the top 20 most differentially expressed genes between samples (pupa vs. egg & nymph; and adult vs. pupa) (Additional file 9). We found that more than 60% of the highly regulated genes are orphan sequences - no homologues found in the NCBI database. This might be consistent with the expectation that Hemiptera development expresses genes with no homologues in other species.

### Functional annotation of differentially expressed genes

**Figure 8:**
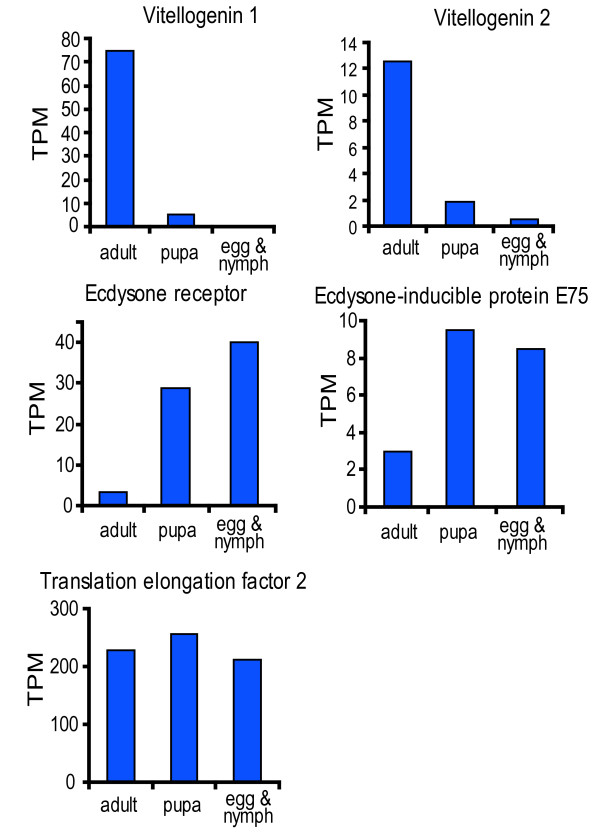
**Analyses of differentially expressed genes during whitefly development**. The gene expression levels of vitellogenin 1 (A); vitellogenin 2 (B); ecdysone receptor (C); ecdysone-inducible protein E75 (D) and translation elongation factor 2 (E) were determined by calculating the number of unambiguous tags for each gene and then normalizing to TPM (transcript copies per million tags).

To understand the functions of differentially expressed genes, we mapped all the genes to terms in KEGG database and, compared this with the whole transcriptome background, with a view to search for genes involved in metabolic or signal transduction pathways that were significantly enriched. Among all the genes with KEGG pathway annotation, 284 differentially expressed genes were identified between pupa and egg & nymph libraries. Notably, specific enrichment of genes was observed for pathways involved in energy and lipid metabolism, such as the citrate cycle, phenylpropanoid biosynthesis and fatty acid metabolism. In the citrate cycle pathway, all the differentially expressed genes were significantly upregulated in pupa, including pyruvate dehydrogenase, isocitrate dehydrogenase, pyruvate carboxylase and citrate synthase. This probably indicates that the metabolic rate of whitefly at pupa stage is higher than that of whitefly at egg & nymph stages. Between adult and pupa stages, a total of 514 differentially expressed genes were found and a specific enrichment of starch and sucrose metabolism pathways was noticed. Interestingly, we also found the enrichment of differentially expressed genes in the glycosphingolipid biosynthesis pathway. To further evaluate our DGE data, we analyzed the expression level of vitellogenin which is an egg yolk gene highly expressed in the adult female [30]. As shown in Figure 8A-B, both vitellogenin 1 and vitellogenin 2 were highly expressed in the adult whitefly. However, both genes were expressed at significantly lower levels in the pupa stage and only vitellogenin 2 is present during the egg & nymph stage. For ecdysone receptor and ecdysone-inducible protein E75, we noticed a much high expression level in nymphs and pupas than that in adult whitefly (Figure 8C-D). This is consistent with their roles in the orchestration of development during molts and metamorphosis [31]. As a control, the translation elongation factor 2 is equally expressed during the three developmental stages (Figure 8E).

## Conclusion

With this study, we present a rapid and cost-effective method for transcriptome and DGE analysis using Illumina sequencing technology. The single run produced more than 168,900 distinct sequences with 27,290 sequences having an above cut-off BLAST result. These findings provide a substantial contribution to existing sequence resources for the whitefly and will certainly accelerate insecticide resistance research in the Q biotype whitefly. To our knowledge, this is the first publication using Illumina sequencing technology for an organism without prior genome annotation. Additionally, we have demonstrated the feasibility of using Illumina sequencing based DGE system for gene expression profiling and have provided new leads for functional studies of genes involved in whitefly development.

## Methods

### Insect rearing and sample preparation

Stock cultures of Q biotype whiteflies (*Bemisia tabaci*) (cytochrome oxidase I sequence GenBank accession no: DQ473394) were maintained on cotton *Gossypium hirsutum *L (Malvaceae) cv. "Zhe-Mian 1793" in climate chambers at 27 ± 1°C, a photoperiod of 14 h light:10 h darkness and 70 ± 10% relative humidity. The purity of the cultures was monitored every 3-5 generations using the random amplified polymorphic DNA-polymerase chain reaction technique and the sequence of mitochondrial cytochrome oxidase I gene, which has been used widely to differentiate whitefly (*B. tabaci*) "biotypes" [32]. Since the quantity of eggs is extremely low, a mixture of eggs and first to third nymphs were collected as one sample. The pupae were collected as another sample. For adults, individuals were collected from the culture using a glass tube (5 × 0.5 cm) and the sex was determined under a stereo microscope. Then adults of the same sex were pooled into a plastic tube using an aspirator. Finally, these samples were frozen at -80°C until use.

### RNA isolation and library preparation for transcriptome analysis

Total RNA was isolated using SV total RNA isolation system (Promega) according to the manufacturer's protocol. RNA integrity was confirmed using the 2100 Bioanalyzer (Agilent Technologies) with a minimum RNA integrated number value of 8. The samples for transcriptome analysis were prepared using Illumina's kit following manufacturer's recommendations. Briefly, mRNA was purified from 6 μg of total RNA (a mixture of RNA from egg & nymph, pupa and adult at equal ratio) using oligo (dT) magnetic beads. Following purification, the mRNA is fragmented into small pieces using divalent cations under elevated temperature and the cleaved RNA fragments were used for first strand cDNA synthesis using reverse transcriptase and random primers. This was followed by second strand cDNA synthesis using DNA polymerase I and RNaseH. These cDNA fragments then went through an end repair process and ligation of adapters. These products were purified and enriched with PCR to create the final cDNA library.

### Analysis of Illumina sequencing results

The cDNA library was sequenced on the Illumina sequencing platform (GAII). The size of the library is approximately 200 bp and both ends of the libraries are sequenced. Image deconvolution and quality value calculations were performed using the Illumina GA pipeline 1.3. The raw reads were cleaned by removing adaptor sequences, empty reads and low quality sequences (reads with unknown sequences 'N'). The reads obtained were randomly clipped into 21 bp K-mers for assembly using de Bruijn graph and SOAPdenovo software [23]. After assessing different K-mer sizes, we found that the 21-mer provided the best result for transcriptome assembly. Small K-mers make the graph very complex; while large K-mers can have poor overlap in regions with low sequencing depth. After sequence assembly, the resultant contigs were joined into scaffolds using the read mate pairs. To obtained distinct gene sequences, the scaffolds were clustered using TGI Clustering tools [24]. Distinct sequences were used for blast search and annotation against an NCBI nr database using an E-value cut-off of 10^-5^. Functional annotation by gene ontology terms (GO; http://www.geneontology.org) was analyzed by Blast2go software. The COG and KEGG pathways annotation was performed using Blastall software against Cluster of Orthologous Groups database and Kyoto Encyclopedia of Genes and Genomes database. The data sets are available at the NCBI Short Read Archive (SRA) with the accession number: **SRX018661**. The assembled sequences have been deposited in the NCBI's TSA database and can be searched using the Gene-ID listed in Additional file 2.

### Digital gene expression library preparation and sequencing

Tag library preparation for the three Q biotype whitefly samples (egg & nymph, pupa, and adult) was performed in parallel using Illumina gene expression sample preparation kit. Briefly, total RNA from the three samples was used for mRNA capture with magnetic oligo(dT) beads. First and second strand cDNA were synthesized and bead-bound cDNA was subsequently digested with NlaIII. The cDNA fragments with 3' ends were then purified with magnetic beads and Illumina adapter 1 was added to their 5' ends. The junction of Illumina adapter 1 and CATG site is the recognition site of MmeI, which cuts 17 bp downstream of the CATG site, producing tags with adapter 1. After removing 3' fragments with magnetic beads precipitation, Illumina adapter 2 was introduced at 3' ends of tags, acquiring tags with different adapters at both ends to form a tag library. After 15 cycles of linear PCR amplification, 85 base strips were purified by PAGE gel electrophoresis. These strips were then digested, and the single-chain molecules were fixed onto the Illumina sequencing chip for sequencing. The data sets are available at the NCBI SRA with the accession number: **SRX018662**.

### Analysis and mapping of DGE tags

Sequencing-received raw image data was transformed by base calling into sequence data. Prior to mapping reads to the reference database, we filtered all sequences to remove adaptor sequence, low quality sequences (tags with unknown sequences 'N'), empty tags (sequence with only adaptor sequences but no tags); low complexity, and tags with a copy number of 1 (probably sequencing error). A preprocessed database of all possible CATG+17 nucleotide tag sequences was created using our transcriptome reference database. For annotation, all tags were mapped to the reference sequences and only allowed no more than 1 nucleotide mismatch. All the tags mapped to reference sequences from multiple genes were filtered and the remaining tags were designed as unambiguous tags. For gene expression analysis, the number of expressed tags was calculated and then normalized to TPM (number of transcripts per million tags); and the differentially expressed tags were used for mapping and annotation. The complete lists of differentially expressed genes are shown in Additional file 7 and 8.

### Evaluation of DGE libraries

A statistical analysis of the frequency of each tag in the different cDNA libraries was performed to compare gene-expression in different developmental stages. Statistical comparison was performed with a custom written scripts using the method described by Audic *et al. *[29]. FDR (false discovery rate) was used to determine the threshold of P value in multiple test and analysis. We used FDR < 0.001 as the threshold to judge the significance of gene expression difference. For pathway enrichment analysis, we mapped all differentially expressed genes to terms in KEGG database and looked for significantly enriched KEGG terms compared to the genome background.

## Authors' contributions

SSL, CXZ and JBL conceived and designed the experimental plan. JBL, JML and YYB performed experiments. XWW and JBL analyzed and interpreted the sequence data. XWW, JBL, CXZ and SSL drafted the manuscript. All authors read and approved the final manuscript.

## Supplementary Material

Additional file 1 **Overview of Q-biotype whitefly transcriptome sequencing and assembly**. (A) Size distribution of Illumina sequencing contigs. (B) Size distribution of distinct sequences after paired-end and gap filling. Click here for file

Additional file 2 **Top BLAST hits from NCBI nr database**. BLAST results against the NCBI nr database for all the distinct sequences with a cut-off E value above 10^-5 ^are shown. Click here for file

Additional file 3 **Predicted amino acid sequence of Singletons2670 which is homologous to insect acetylcholinesterase 1 (AChE1) and alignment with B biotype whitefly AChE1**. The three point mutations (amino acid: 64, 68 and 233) are show in light blue. Click here for file

Additional file 4 **Correlation analysis of DGE libraries**. The correlation between pupa and egg & nymph libraries; adult and pupa libraries are shown. Dots in the figures indicate individual tag entities. Pearson correlation coefficients are shown in the upper left corner of each plot. Click here for file

Additional file 5 **Relationship between the number of detected genes and sequencing amount (total tag number)**. All figures show a trend of saturation. When the sequencing amount reaches 2 millions, the number of detected genes almost ceases to increase. Click here for file

Additional file 6 **Summary of the most abundant genes expressed in adult whiteflies with annotation**. TPM: number of transcripts per million tags. Click here for file

Additional file 7 **Differentially expressed genes between adult and pupa**. TPM: transcript copies per million tags. Raw intensity: the total number of tags sequenced for each gene. FDR: false discovery rate. We used FDR < 0.001 and the absolute value of log2Ratio ≤1 as the threshold to judge the significance of gene expression difference. In order to calculate the log2Ratio and FDR, we used TPM value of 0.001 instead of 0 for genes that do not express in one sample. Click here for file

Additional file 8 **Differentially expressed genes between pupa and egg & nymph**. TPM: transcript copies per million tags. Raw intensity: the total number of tags sequenced for each gene. FDR: false discovery rate. We used FDR < 0.001 and the absolute value of log2Ratio ≤1 as the threshold to judge the significance of gene expression difference. In order to calculate the log2Ratio and FDR, we used TPM value of 0.001 instead of 0 for genes that do not express in one sample. Click here for file

Additional file 9 **The top 20 most up-regulated and down-regulated genes between samples (pupa vs. egg & nymph; adult vs. pupa)**. TPM: transcript copies per million tags. This table does not include genes that only expressed in one sample. Click here for file
