# Supplementary figures and images for: De novo characterization of a whitefly transcriptome and analysis of its gene expression during development
Source: BMC Genomics. 2010 Jun 24;11:400. doi: 10.1186/1471-2164-11-400 (PMC2898760; doi:10.1186/1471-2164-11-400)

A

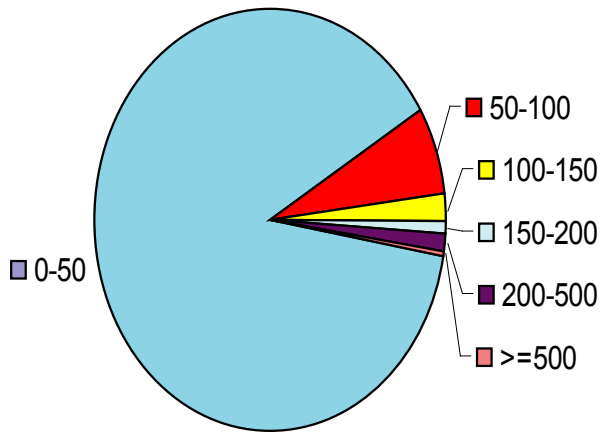

B

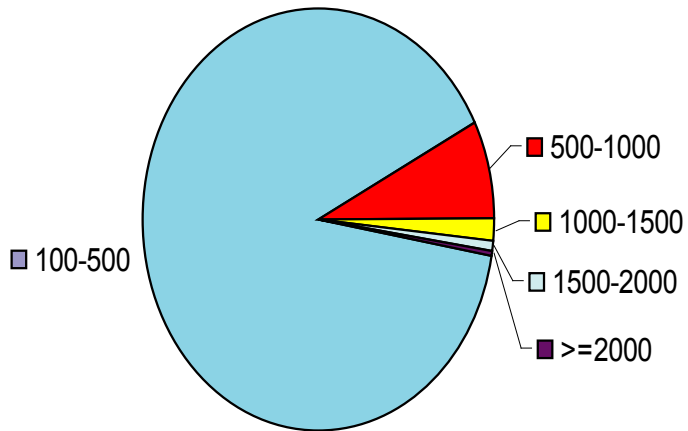

Supplement: Additional file 1 — Overview of Q-biotype whitefly transcriptome sequencing and assembly. (A) Size distribution of Illumina sequencing contigs. (B) Size distribution of distinct sequences after paired-end and gap filling. [file 1471-2164-11-400-S1.PDF]

pupa vs. egg & nymph

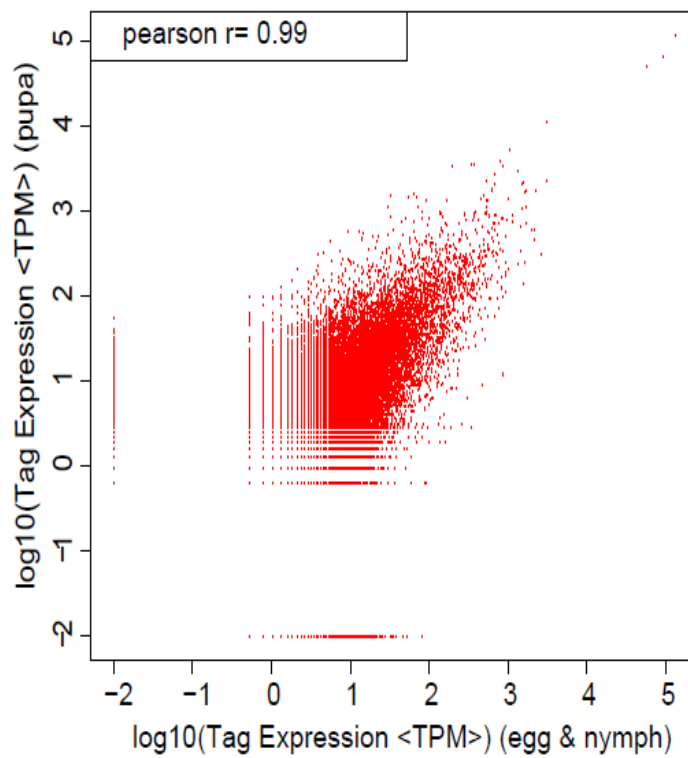

adult vs. pupa

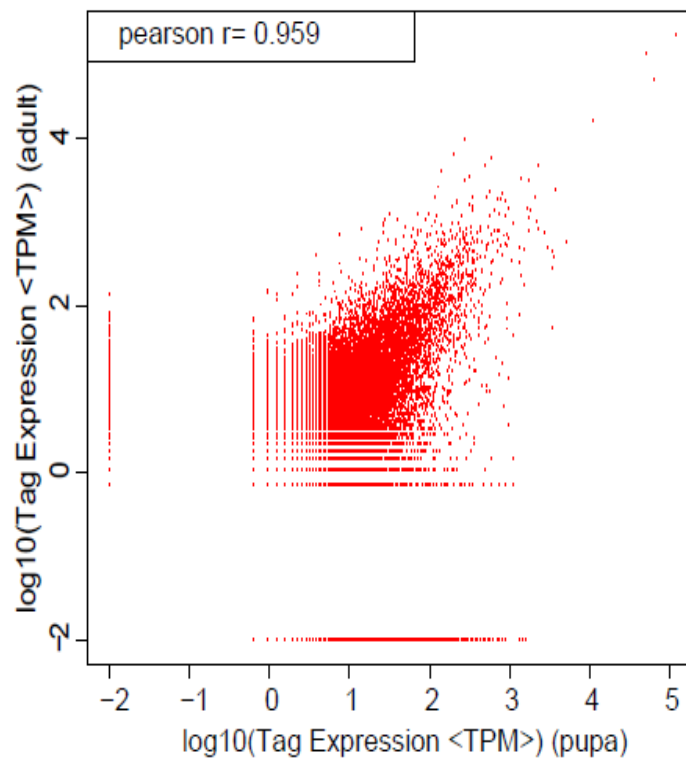

Supplement: Additional file 4 — Correlation analysis of DGE libraries. The correlation between pupa and egg & nymph libraries; adult and pupa libraries are shown. Dots in the figures indicate individual tag entities. Pearson correlation coefficients are shown in the upper left corner of each plot. [file 1471-2164-11-400-S4.PDF]

## Egg & nymph

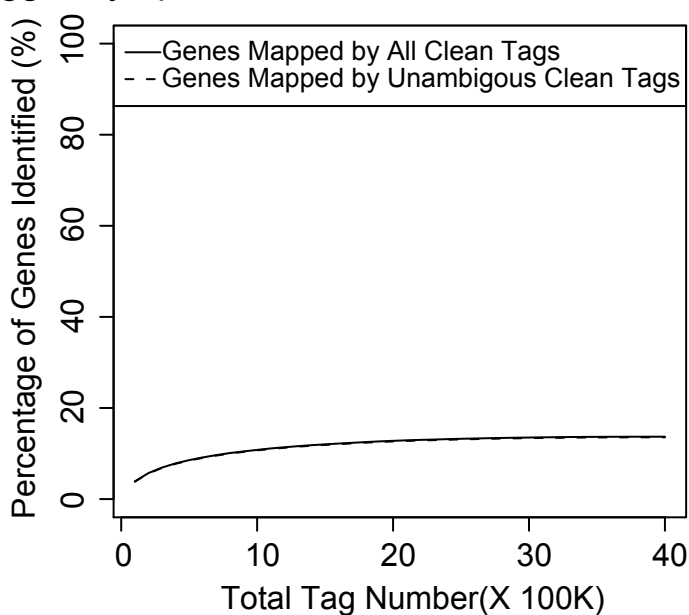

## Pupa

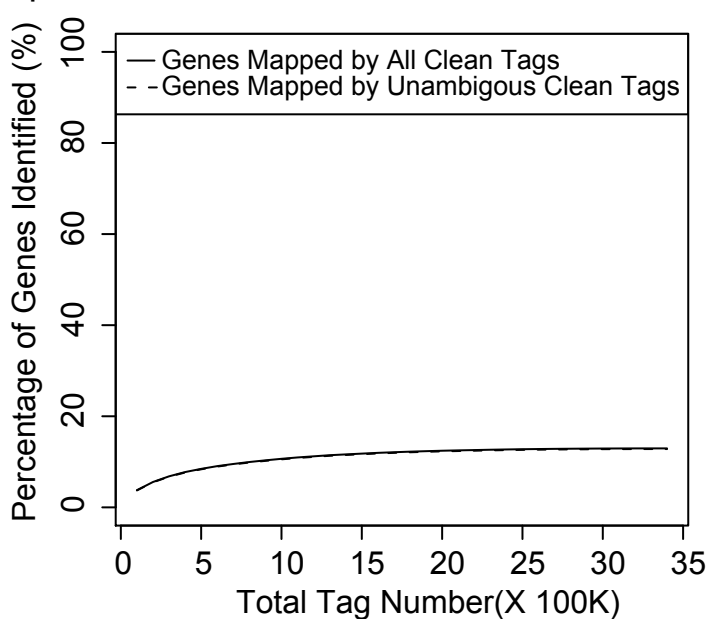

## Adult

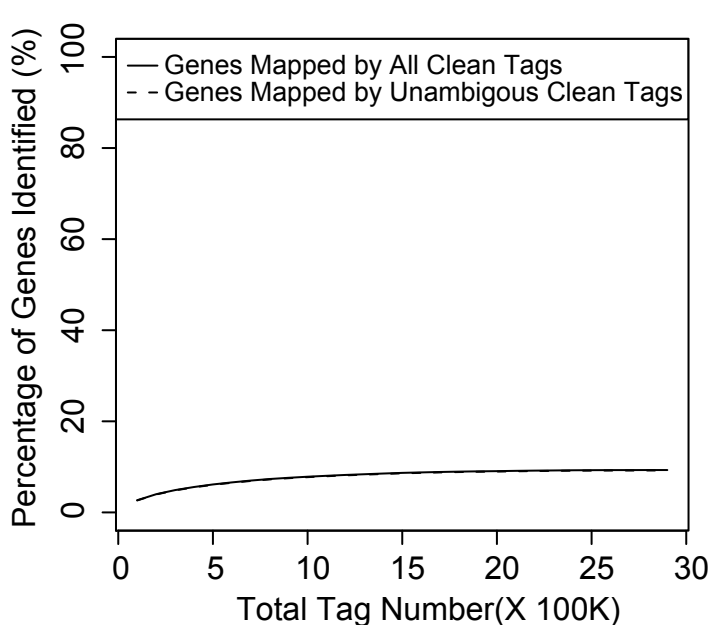

Supplement: Additional file 5 — Relationship between the number of detected genes and sequencing amount (total tag number). All figures show a trend of saturation. When the sequencing amount reaches 2 millions, the number of detected genes almost ceases to increase. [file 1471-2164-11-400-S5.PDF]
