# Supplementary material for: De novo characterization of a whitefly transcriptome and analysis of its gene expression during development
Source: BMC Genomics. 2010 Jun 24;11:400. doi: 10.1186/1471-2164-11-400 (PMC2898760; doi:10.1186/1471-2164-11-400)
Supplement: Additional file 3 — Predicted amino acid sequence of Singletons2670 which is homologous to insect acetylcholinesterase 1 (AChE1) and alignment with B biotype whitefly AChE1. The three point mutations (amino acid: 64, 68 and 233) are show in light blue. [file 1471-2164-11-400-S3.PDF]

```
Singletons2670      MDFDHLPLRASPETDQLRNPRHGFGFRDGISDEGLNFRHSEHEGERSKYK   50
B-biotype AChE1     MDFDHLPLRASPETDQLRNPRHGFGFRDGISDEGLNFRHSEHEGERSKYK   50

Singletons2670      GAEAEEEMMADEGDKDPLGVQTTKGKVRGTTLTAATGKQVDAWLGIPYAQ   100
B-biotype AChE1     GAEAEEEMMADEGDNDPLVVQTTKGKVRGTTLTAATGKQVDAWLGIPYAQ   100

Singletons2670      KPIGALRFRHPRPIDKWEGILNATKMPNSCTQIVDTVFGDFAGSAMWNPN   150
B-biotype AChE1     KPIGALRFRHPRPIDKWEGILNATKMPNSCTQIVDTVFGDFAGSAMWNPN   150

Singletons2670      TPMSEDCLYINVITPKPRPRNAAVMVWIFGGGFYTGTATLDIYDYKILAS   200
B-biotype AChE1     TPMSEDCLYINVITPKPRPRNAAVMVWIFGGGFYTGTATLDIYDYKILAS   200

Singletons2670      EENVILVSMQYRITCLGFLYFDTQDVPGNAGLFAQLMALQWIRNNIHAFG   250
B-biotype AChE1     EENVILVSMQYRITCLGFLYFDTQDVPGNAGLFDQLMALQWIRNNIHAFG   250

Singletons2670      GNPHNITLFGESAGA                                      265
B-biotype AChE1     GNPHNITLFGESAGA                                      265
```
