# Supplementary material for: De novo characterization of a whitefly transcriptome and analysis of its gene expression during development
Source: BMC Genomics. 2010 Jun 24;11:400. doi: 10.1186/1471-2164-11-400 (PMC2898760; doi:10.1186/1471-2164-11-400)
Supplement: Additional file 6 — Summary of the most abundant genes expressed in adult whiteflies with annotation. TPM: number of transcripts per million tags. [file 1471-2164-11-400-S6.DOC]

**Summary of the most abundant genes expressed in adult whiteflies**

| **Number** | **Gene ID** | **TPM** | **Gene description** |
| --- | --- | --- | --- |
| 1 | Singletons87059 | 518.54 | Glyceraldehyde-3-phosphate dehydrogenase |
| 2 | Singletons5080 | 381.4 | Tubulin alpha chain |
| 3 | Singletons8347 | 304.67 | Cytochrome P450 CYP6CX1v2 |
| 4 | Singletons138628 | 225.73 | Translation elongation factor 2 |
| 5 | Singletons814 | 224.61 | Lysosomal alpha-mannosidase |
| 6 | Singletons11510 | 223.5 | Cathepsin L 1 |
| 7 | Singletons25445 | 206.45 | GI24842 [Drosophila mojavensis] |
| 8 | Singletons73306 | 170.5 | 40S ribosomal protein S15 |
| 9 | Singletons11953 | 167.53 | 28S ribosomal protein S35, mitochondrial |
| 10 | Singletons21991 | 151.97 | ATP-dependent RNA helicase |
| 11 | Singletons22399 | 146.41 | BTB/POZ domain-containing protein 10 |
| 12 | Singletons22843 | 130.84 | 2-acylglycerol O-acyltransferase 3 |
| 13 | Singletons22499 | 124.17 | Tuberin |
| 14 | Singletons7863 | 120.46 | Nrglycerophosphoryl diester phosphodiesterase |
| 15 | Singletons2644 | 110.82 | Basic salivary proline-rich protein 1 |
| 16 | Singletons4823 | 107.12 | Ferrochelatase |
| 17 | Singletons8929 | 97.85 | COP9 signalosome complex subunit |
| 18 | Singletons10574 | 95.26 | Thioredoxin domain-containing protein 12 |
| 19 | Singletons15229 | 94.52 | Developmentally-regulated GTP-binding protein |
| 20 | Singletons5219 | 91.18 | Hypothetical protein |

TPM: number of transcripts per million tags.
